# Unyielding hearts: parental adaptation and resilience among families of children with autism in China

**DOI:** 10.3389/fpsyg.2026.1826241

**Published:** 2026-06-24

**Authors:** Ling Chen, Hui Zhang, Jiahui Tian, Xinyi Zhang

**Affiliations:** 1 School of Law and Economics, Wuhan University of Science and Technology, Wuhan, China; 2 School of Sociology, Central China Normal University, Wuhan, China; 3 School of Law, Jianghan University, Wuhan, China

**Keywords:** adaptation trajectory, adaptive strategy, China, cultural context, family dynamics, parents of children with autism, resilience, social support

## Abstract

**Introduction:**

Autism Spectrum Disorder (ASD) is a lifelong neurodevelopmental condition characterized by persistent social communication difficulties, repetitive behaviors, and restricted interests. Although interventions may alleviate some symptoms, ongoing challenges in treatment, education, and social inclusion impose substantial physical and emotional burdens on parents, particularly in contexts with limited resources, such as China. Understanding parental positive adaptation is therefore critical for promoting family well-being.

**Methods:**

This qualitative study employed in-depth interviews and participant observation with 16 full-time caregivers of children with ASD (aged 0–18 years) in Wuhan, China, to examine their everyday experiences and adaptive processes. Guided by resilience theory, data were analyzed using situational and categorical analyses.

**Results:**

We identified motivational sources at individual, family, and societal levels, as well as adaptive strategies among successfully adapted parents, including active self-regulation, diverse social support networks, and proactive community engagement.

**Discussion:**

This study contributes a culturally grounded resilience framework for families of Chinese children with ASD and provides implications for social work practice, autism intervention, and policy development aimed at enhancing parental adaptation.

## 1 Introduction

Autism Spectrum Disorder (ASD) is a neurodevelopmental disorder characterized by persistent deficits in social interaction, communication, and restricted, repetitive behaviors (American Psychiatric Association, 2013). Although intervention services can alleviate certain symptoms, ASD remains a lifelong, incurable condition. Recent estimates indicate a 1% prevalence of ASD in China, consistent with global trends (Wucailu Autism Research Institute, 2017), corresponding to over 10 million individuals on the spectrum, including more than two million children aged 0–14 years. In line with rising global prevalence, China faces a growing population of individuals with ASD, whose families encounter substantial challenges in managing long-term care demands.

Autism intervention requires sustained financial and caregiving commitments. During the critical early intervention period, effective treatment often involves frequent and long-term rehabilitation sessions, placing substantial and prolonged economic strain on most working- and middle-class families. At the same time, intensive parental involvement is typically necessary, leading many caregivers to suspend or abandon paid employment to focus on their child’s care (Huang et al., 2024). Although such dedication may improve intervention outcomes, it simultaneously reduces household income, creating a persistent cycle of high expenditure and declining earnings.

Caring for a child with ASD is highly demanding, yet some parents successfully adapt to adversity and gradually regain stability through proactive coping. Based on in-depth interviews with 16 parents, in this study we found that participants experienced cyclical psychological fluctuations, moving from periods of despair to initial adaptation and subsequent setbacks, resulting in two distinct long-term trajectories: persistent distress or sustained resilience. This divergence informs two core research questions. The first asks, under comparable adversities, what internal motivational mechanisms enable some parents to maintain psychological resilience. The second asks what specific adaptive strategies characterize parents who successfully achieve positive adaptation.

Against the backdrop of global efforts in inclusive positive psychology interventions for vulnerable populations, families of children with ASD represent a key marginalized group with substantial unmet mental health and support needs. In China, limited institutional support, cultural norms of family-centered care, and social stigma exacerbate the challenges faced by these parents, making the study of their resilience and adaptive processes essential for informing inclusive interventions. In this study, we address the research theme of Inclusive Positive Psychology Interventions for Vulnerable Groups: Bridging Gaps and Fostering Resilience by identifying culturally specific resilience mechanisms and proposing targeted support strategies for families of Chinese children with ASD, thereby contributing to the global evidence base on positive psychology interventions for marginalized caregivers.

## 2 Literature review

### 2.1 Research on parents of children with autism

Research on parents of children with autism has long focused on the psychological and social challenges they face, consistently documenting substantial caregiver burden. Caring for an autistic child significantly increases the risk of parental depression and anxiety and reduces overall quality of life (Alenezi et al., 2024). Parents of children with autism and intellectual disabilities often report persistent mental health difficulties related to concerns about their children’s future, financial strain, and stigma (Xiao and Sun, 2019; Mak and Kwok, 2010; Ljungberg, 2025). Although this body of research has yielded important insights into parental vulnerability, its deficit-oriented focus, together with the expectation that parents serve as the primary force in their child’s development, may inadvertently heighten psychological pressure, particularly among mothers (Dijkstra-de Neijs et al., 2024). Moreover, deficit frameworks tend to obscure parents’ coping resources and adaptive strengths, limiting the development of effective family-centered interventions (Zhao, 2021).

In response, resilience theory has gained prominence as a strength-based perspective that emphasizes parents’ psychological resources and adaptive potential. Social support and resilience are mutually reinforcing, with active coping serving as a key mechanism linking the two (Wang et al., 2025). Empirical studies further show that enhancing mothers’ problem-solving abilities and psychological capital promotes their well-being and may positively influence their autistic children (Al Eid et al., 2024). Key resilience factors include socioeconomic resources, social support, open family communication, a supportive family environment, family hardiness, flexible coping strategies, a positive outlook on life, and shared family belief systems (Greeff and van der Walt, 2010).

Beyond the focus on psychological burden and resilience, scholars have also examined parental adaptation through stage-based models. From a family life-cycle perspective, Lu et al. (2014) propose six stages in the adaptation of families with autistic children, namely formation, confusion, diagnosis, identity recognition, caregiving, and family fragmentation. While this framework outlines the overall trajectory of family experiences, it offers limited insight into parents’ agency and subjective meaning-making. A related five-stage psychological adjustment model, comprising shock, denial, confusion, hope, frustration, and acceptance, has also been widely cited (Pressman, 1987). This model foregrounds intrapsychic adjustment but pays comparatively little attention to contextual conditions and everyday caregiving practices. Gou (2024) further distinguishes three major adaptation stages corresponding to early childhood, school age, and adulthood, emphasizing shifts in parental responsibilities and emotional trajectories across the life course. Despite differences in classification, these studies consistently demonstrate that parental adaptation is a dynamic and fluctuating process shaped by changes in psychological states, family circumstances, and external support systems.

Coping strategies constitute another important analytical lens for understanding parental adaptation. Coping responses are commonly classified into problem-focused coping, which targets the source of stress, and emotion-focused coping, which aims to regulate negative emotional states (Folkman et al., 1986). However, this dichotomy is limited by its broadness, as parents often employ both strategies concurrently in response to complex situations. Studies on parents of children with autism have also distinguished between positive and negative coping based on emotional orientation (Pottie and Ingram, 2008), yet this typology is constrained by the lack of a unified standard for defining emotional states. Moreover, little is known about when and why parents shift between different coping strategies, or how such shifts shape long-term adaptation. These gaps point to the need for process-oriented analyses that capture the dynamics of parental coping over time.

In recent years, autism intervention has increasingly extended beyond the family sphere, calling for broader societal engagement and interdisciplinary collaboration. As an applied discipline grounded in systematic theory and practice, social work holds particular potential in supporting individuals with developmental disabilities by delivering direct services and mobilizing community and institutional resources for environmental support (Xu and Wang, 2020). However, research that traces the long-term developmental trajectories of challenging behaviors and integrates insights across professional fields remains limited (Adams et al., 2023). Differences in theoretical frameworks and research paradigms continue to impede cross-disciplinary communication, yet the complementarity among disciplines also underscores the substantial potential for future collaborative intervention and research (Cascio et al., 2016).

A review of the literature reveals a clear shift from a problem-focused orientation toward strength-based and resilience-oriented perspectives (Parker et al., 2020). However, the substantive scope of current research remains limited, particularly in domestic scholarship, which has paid insufficient attention to the mechanisms, motivations, and concrete strategies underlying parental adaptation. Although some resilience-related factors have been identified, parents’ deeper psychological motivational processes remain underexplored. Methodologically, the dominance of quantitative approaches captures overall patterns but often overlooks the complexity of lived experiences, while the relative lack of qualitative research constrains insight into parents’ meaning-making. Disciplinary contributions are likewise uneven, with psychiatry, psychology, and education dominating the field, and social work remaining weakly integrated. Together, these gaps underscore the need for more multi-perspective and empirically grounded research on parental adaptation in the context of autism.

### 2.2 Review of resilience theory

Resilience is commonly defined as the capacity to maintain or rapidly regain psychological well-being in the face of adversity (Bonanno et al., 2011; Kalisch et al., 2017). As a core strength-based paradigm in psychology and sociology, resilience theory emphasizes individuals’ active use of internal resources and external support to cope with stress and achieve positive adaptation (Tian, 2007; Jia, 2001). Western scholarship generally conceptualizes resilience around three elements: exposure to adversity, the mobilization of protective resources, and the attainment of adaptive outcomes (Liu, 2022). Accordingly, resilience is understood as a dynamic process shaped by the interaction of individual characteristics, environmental contexts, and social support systems (Kalisch et al., 2019). The Individual and Environmental Resilience Model further maps these protective factors across multiple domains, including family, school, peers, culture, and community, together with individual biological, cognitive, emotional, and behavioral capacities that jointly buffer the effects of adversity (Llistosella et al., 2022).

Research indicates that both direct and indirect family support plays a central role in the development of individual resilience and constitutes an independent functional component in resilience formation (Feng, 2014; Cheng et al., 2024). However, conceptualizing family resilience solely at the household level and assessing it through individual family members limits its analytical precision (An et al., 2023). Family resilience is further shaped by household background, socioeconomic status, family structure, and the interaction among these factors (Nadrowska et al., 2022). Western theories of family resilience are largely grounded in individualistic cultural assumptions (An et al., 2023), whereas in China, individuals typically depend on family-based and extended social networks when coping with stress, reflecting the “differential mode of association” (Yan, 2017). This cultural divergence suggests that direct transplantation of Western family resilience theories may lead to conceptual misalignment, highlighting the need for culturally grounded theoretical adaptation that emphasizes multi-layered support systems and ethically embedded familial ties (An et al., 2023; Tao, 2023).

Based on these considerations, in this study we adopt the developmental processes and operating mechanisms of individual resilience as the primary analytical lens for examining how parents of children with autism adapt to ongoing challenges. At the same time, within the Chinese cultural context, family resilience theory is used as a complementary perspective to offer a more comprehensive understanding of the motivational sources and adaptive strategies that shape parental adjustment.

## 3 Methods

### 3.1 Study design and participants

Using a qualitative research design, in this study we employed in-depth interviews and participant observation to examine parents’ adaptation trajectories from diagnosis onward and to explore the motivations and strategies underlying their coping processes. Participants were full-time caregivers of children with ASD aged 0–18 years in Wuhan, China. Wuhan was selected as the research site because it is a major central city with a growing ASD service system and diverse socioeconomic contexts, providing a representative sample of urban Chinese families.

Participants were recruited through three channels: a professional autism rehabilitation center, a parent-led mutual-aid organization, and the social media platform Xiaohongshu (Rednote). Snowball sampling with multiple entry points was used to reduce sampling bias and ensure diversity in caregiver characteristics, including gender, age, educational background, and child ASD severity. In total, 16 parents were interviewed (Table 1). The sample size was determined by thematic saturation, as no new themes or insights emerged after the 15th interview, confirming the adequacy of the qualitative data for analysis (Braun and Clarke, 2006).

**Table 1:** Demographic characteristics of interviewees (*N* = 16).

Number	Gender	Age	Occupation	Educational background	Marital status
A1	Female	40	Stay-at-home mother	Master’s degree	Married
A2	Female	42	Stay-at-home mother	Bachelor’s degree	Married
A3	Female	29	Stay-at-home mother	Bachelor’s degree	Married
A4	Male	38	Entrepreneur	Bachelor’s degree	Married
A5	Female	36	Stay-at-home mother	Bachelor’s degree	Married
A6	Male	36	IT Programmer	Bachelor’s degree	Married
A7	Male	55	Director, parent-led autism support organization	Bachelor’s degree	Divorced
A8	Female	52	Physician	Bachelor’s degree	Married
A9	Female	32	Elementary school teacher	Bachelor’s degree	Married
A10	Female	52	Contractual urban-management officer	Junior High School	Divorced
A11	Female	37	UI Designer	Bachelor’s degree	Married
A12	Female	44	Company employee	Master’s degree	Married
A13	Female	40	Chef	Bachelor’s degree	Married
A14	Female	36	Stay-at-home mother	Bachelor’s degree	Married
A15	Female	54	Director, Rehabilitation Center	Bachelor’s degree	Divorced
A16	Female	39	Stay-at-home mother	Bachelor’s degree	Married

All participants provided written informed consent prior to participation and were informed of the study’s purpose, procedures, and their right to withdraw at any time without penalty. Pseudonyms were assigned to both interviewees and their children, and all identifying information, including names, specific addresses, and rehabilitation center details, was removed to ensure confidentiality.

### 3.2 Data collection

Data were collected between June and December 2024 using semi-structured in-depth interviews and participant observation, with triangulation of data sources to enhance trustworthiness and validity (Creswell and Creswell, 2018). Interviews were conducted face-to-face in quiet, private spaces such as coffee shops or participants’ homes, or online via video call platforms such as WeChat, and lasted 60–120 min each. The interview guide, developed based on resilience theory and existing literature on ASD parental adaptation, served only as a framework for discussion rather than a fixed questionnaire. Core questions were phrased as open-ended prompts to avoid suggesting specific answers or imposing value judgments, allowing participants to guide the narrative according to their own experiences. For example, participants were asked, “Please describe the changes in your life and that of your family before and after your child’s diagnosis.” Participants were free to choose the timeframe, types of events, and emotional dimensions they wished to discuss. This design ensured that the interviews were participant-led and that the data reflected parents’ perspectives rather than assumptions of the researchers. All interviews were audio-recorded with permission and transcribed verbatim into Chinese within 24 h of completion.

The researcher participated as a volunteer in autism rehabilitation center activities, parent mutual-aid gatherings, and online parent discussion groups for 6 months. The researcher’s identity and research purpose were fully disclosed to participants in these settings. Field notes documented everyday interactions, caregiving practices, social support dynamics, and contextual factors influencing parental adaptation. Observation data complemented interview findings by capturing real-world behaviors and social processes that participants may not have articulated in interviews.

In this study we adopted a perspective grounded in social work and positive psychology, which focuses attention on support systems and parents’ potential for positive adaptation, but may also introduce bias toward a “problem–intervention” focus. To address this, a reflective journal was maintained throughout the 6-month fieldwork, documenting key moments such as emotional resonance during interviews, intuitive labeling of segments as positive transformations, and cognitive conflicts arising from data that did not align with resilience theory (e.g., A10’s experience of divorce, A14’s state of numbness). Regular supervision discussions were conducted to differentiate patterns emerging from the data from those influenced by researcher expectations. These procedures ensured transparency, reflexivity, and methodological rigor, providing strong support for the inductive grounding of codes and the development of core themes.

### 3.3 Data analysis

Data were analyzed using situational analysis and categorical analysis, guided by resilience theory, with the qualitative data analysis software NVivo 15 used for coding and theme development. The analysis followed a four-step process (Braun and Clarke, 2006):

The researcher read and re-read all interview transcripts and field notes to develop a comprehensive understanding of the data, marking initial impressions and key insights. Open coding was conducted to label and categorize all meaningful units of data, such as “diagnosis shock,” “financial strain,” “family support,” and “active self-regulation,” generating a total of 216 initial codes. These codes were grouped into broader conceptual categories and refined into core themes and sub-themes. For instance, the codes “parental responsibility,” “optimistic disposition,” and “self-awareness” were combined into the core theme “Individual-level motivational sources of resilience.” To ensure that the themes were grounded in the data rather than influenced by theoretical assumptions, two independent qualitative research experts reviewed the coding, confirming that it captured the diversity of participants’ experiences. Inter-coder agreement was 0.89, demonstrating high reliability. Theoretical interpretation was applied only after the themes were fully developed, preserving the inductive nature of the analysis.

Situational analysis was employed using situational maps and observation notes to capture how parental adaptation interacts with structural and institutional factors. The maps identified key individuals, organizations, and discursive elements that shape adaptation. For example, one map documented actors including rehabilitation center staff, Disabled Persons’ Federation personnel, school homeroom teachers, parent support group members, and university volunteers, showing how parents navigate this network to access resources. Observation notes recorded parents’ interactions and nonverbal behaviors during activities at mutual aid organizations and rehabilitation centers, providing behavioral validation for the interview data. Together, these methods ensured that the themes reflected both participants’ experiences and the broader structural context, strengthening the credibility and explanatory power of the analysis.

### 3.4 Demographic characteristics of participants

Table 1 presents the demographic characteristics of the 16 interviewees, and Table 2 presents the characteristics of their children with ASD. The sample included 13 mothers and 3 fathers (reflecting the gendered caregiving norm for children with ASD in China), with ages ranging from 29 to 55 years (*M* = 40.63, SD = 7.89). Most participants had a bachelor’s degree (*n* = 15) or master’s degree (*n* = 1), and the majority were married (*n* = 13); three participants were divorced, all of whom were female caregivers. The children with ASD were aged 5–17 years (*M* = 10.81, SD = 3.52), with diagnosis ages ranging from 1.5 to 3 years (*M* = 2.31, SD = 0.47); most had moderate ASD (*n* = 12), with four having moderate-to-severe ASD and one having mild ASD. The majority of children attended mainstream primary/kindergarten schools (*n* = 11), with three in special-education vocational schools, one in a special-education school, and another receiving home-based education.

**Table 2:** Characteristics of children with ASD (*N* = 16).

Number	Gender	Age	Age at ASD diagnosis	ASD severity level	Educational placement
B1	Male	12	2	Moderate	Mainstream primary school
B2	Male	10	1.5	Moderate-to-severe	Mainstream primary school
B3	Male	5	2.5	Moderate	Mainstream kindergarten
B4	Male	9	2	Moderate	Mainstream primary school
B5	Male	10	3	Moderate	Mainstream primary school
B6	Male	8.5	2.5	Moderate	Mainstream primary school
B7	Male	12	1.5	Moderate	Mainstream primary school
B8	Male	17	2	Mild	Special-education vocational school
B9	Female	7	2	Moderate	Mainstream primary school
B10	Female	14	3	Moderate-to-severe	Home-based education
B11	Male	12	3	Mild	Mainstream primary school
B12	Male	12	2	Moderate-to-severe	Mainstream primary school
B13	Male	16	3	Moderate	Special-education vocational school
B14	Male	7	2	Moderate-to-severe	Mainstream primary school
B15	Male	15	1.5	Moderate-to-severe	Special-education school
B16	Female	8	3	Moderate	Mainstream primary school

## 4 Results

### 4.1 Adaptation trajectories of parents of children with ASD: a dynamic, cyclical process

Based on in-depth interviews with 16 parents, we found that most participants experienced cyclical psychological fluctuations, moving from despair to initial adaptation and subsequent setbacks, with long-term adjustment diverging into two trajectories: active adaptation and adaptation difficulty. A small proportion of participants did not exhibit this cyclical pattern; their trajectories were characterized by persistent distress without significant periods of initial adaptation or renewed hope. These patterns were observed in most, but not all participants, highlighting variability in adaptation trajectories.

#### 4.1.1 Collapse and despair: imbalance under multiple stressors

Following an autism diagnosis, parents typically experienced a psychological and life trajectory from shock and collapse toward gradual adaptation. In the initial stages, they simultaneously endured psychological trauma, financial burdens, and strained family relationships. Accumulating pressures often led to weakening of the family support system, creating substantial imbalance.

For most parents, the diagnosis signifies the collapse of their existing social identity. Since children with autism often appear indistinguishable from typically developing peers and autism awareness remains limited, parents frequently harbored wishful thinking before diagnosis, attributing delays in speech, unresponsiveness, or unusual behaviors to individual variation. Hearing the doctor’s diagnosis directly plunges them into denial and profound shock, forcing a transition from “ordinary parent” to “parent of a child with special needs” and engendering a dual collapse of social identity and self-expectations.

“After the diagnosis was confirmed, I could not accept it. It felt like a cruel joke from the heavens. I was utterly helpless.” (Interviewee A5).

Parents had to dismantle their previous “typical parent” identity while simultaneously attempting to rebuild their identity as a “special-needs parent” accepting their child’s developmental path under social stigma and long-term care demands.

Psychological collapse is often only the beginning of parents’ struggles. The more enduring test lies in the long and costly process of rehabilitation. After diagnosis, many parents rush to seize the limited window for early intervention and actively seek treatment. Autism intervention is typically prolonged and financially demanding: professional sessions usually cost 200–500 RMB per hour (approximately 28–70 USD), and effective intervention during early childhood commonly requires more than 10 sessions per week. As a result, monthly expenses can easily reach several thousand or even tens of thousands of RMB, placing a substantial burden on working- and middle-class families. Moreover, intensive rehabilitation demands sustained parental involvement, forcing many parents to quit or suspend their careers. This combination of rising expenditures and declining household income places families under severe and prolonged financial strain.

“At that time, between special education centers, kindergarten, and different rehabilitation institutions, I was constantly running around. I could not even consider going back to work. All my energy and money were invested in my child’s treatment.” (Interviewee A10).

The combined burden of rehabilitation expenses and declining household income was associated with sustained economic strain. Participants reported reduced quality of life, escalating financial pressure, heightened psychological distress, negative emotions, and curtailments in basic living expenditures. These circumstances were linked to weakened coping capacity and increasing family instability.

The accumulation of financial strain not only depletes material resources but also erodes the emotional fabric of the family. Beyond psychological and economic pressures, marital tensions tend to intensify. Under the demands of continuous high-intensity caregiving, which involves managing emotional outbursts, sleep disturbances, and other daily challenges, both partners experience persistent physical and emotional exhaustion. When basic rest and emotional communication become scarce, even minor disagreements can escalate into severe conflicts, including disputes over intervention approaches or educational philosophies.

“We had different worldviews and values, and we clashed over our child’s education. I wanted to invest money in therapy, while he preferred to let things take their natural course, so inevitably we ended up in constant arguments.” (Interviewee A10).

More commonly, marriages in families raising children with autism are strained by patterns of withdrawal from caregiving responsibility. Confronted with lifelong care demands, sustained financial pressure, and uncertain rehabilitation outcomes, one partner may ultimately choose to exit the marriage as a way of escaping these accumulated burdens.

“My husband and I did not have a strong emotional foundation to begin with. When our child had to undergo daily rehabilitation at the hospital, everything became even harder. Facing these circumstances, everyone’s psychological endurance has its limits. I often found myself asking, ‘Why is this happening to me? How am I supposed to go on?’ In the end, my child’s father chose to leave.” (Interviewee A15).

Marital relationships constitute one of the most severe tests for families raising children with autism. While some couples grow closer through shared adversity, many experience escalating strain under long-term caregiving pressure. As tensions accumulate and mutual support weakens, families often descend into helplessness. During prolonged intervention, organizational, financial, temporal, and emotional resources are frequently exhausted, yet children’s progress may remain limited. This sharp gap between sustained effort and uncertain outcomes shatters parents’ hopes for normalization and draws them back into despair. Meanwhile, inadequacies within medical and support systems further aggravate this distress, as standardized treatment often prioritizes medication while failing to address families’ complex psychosocial needs, leaving many parents feeling unsupported and powerless.

“When my child showed no improvement, I fell into a period of deep despair. I felt that life had no future. I was completely lost and hopeless, with no idea where to turn.” (Interviewee A14).

Overall, the phase of collapse and despair reflects the complex and uneven pressures that families encounter in the early stages after diagnosis. Psychological shock, financial strain, and the breakdown of intimate relationships often converge, leaving parents in a state of profound vulnerability and helplessness. Yet it is also under these conditions of near collapse that some parents begin to explore new ways of coping. Through repeated trial and error, they gradually locate sources of support in everyday life and start to gather the emotional and practical resources needed for subsequent initial adaptation.

#### 4.1.2 Initial adaptation: progress through intervention training

Following the phase of collapse and despair, parents gradually rebuild their resolve to pursue systematic interventions for their child. As the child slowly begins to acquire foundational skills through sustained intervention training, these small but tangible improvements provide parents with their earliest sense of positive feedback and renewed hope. This reassurance arises not only from the observable skills themselves, but also from parents’ renewed recognition of their child’s potential. Through these early gains, parents become able to envision their child’s future development in a more realistic and measured way, which in turn facilitates their initial psychological and behavioral adaptation.

“Every new skill is a hurdle, yet every bit of progress is comforting. For example, with bowel movements, he used to stand and defecate in his pants. We used diapers until he was over 5. But we had to teach him to sit. The whole family and his teacher patiently demonstrated [this] hundreds of times. He finally learned to sit on the toilet around age 7 or 8.” (Interviewee A13).

As intervention progresses, gains in language, attention, and rule awareness further bolster parental confidence. Although children with autism may exhibit typical physical development, underlying neurodevelopmental differences present multifaceted challenges, including delayed language development, atypical sensory processing, and lagging social skills. In response, parents carefully select targeted interventions such as speech therapy, sensory-integration training, and behavioral modification, often integrating these practices into daily routines. Through sustained parental effort, children’s abilities improve, which in turn reinforces parents’ confidence to continue their caregiving and intervention efforts.

“He was diagnosed at 18 months, with no language, no attention, and no sense of rules. We enrolled him in numerous intervention programs and practiced with him intensively at home. It was not until he was 4 years and 2 months old that he finally produced his first sound, calling me ‘Dad.’ Although his speech was still unclear, I knew at that moment that he had begun to speak, and I was overwhelmed with joy.” (Interviewee A7).

Parents often bear immense pressure on the journey of raising a child with autism, yet each incremental gain in their child’s development provides substantial psychological relief and renewed confidence. These improvements lead parents to question the prognosis of incurability and begin to adapt to life with their autistic child.

#### 4.1.3 Secondary despair: the resurgence of educational and inclusion pressures

As children reached school age, parents who had initially adapted to the diagnosis reported renewed pressures related to school rejection, limited learning capacity, and difficulties in social integration. These challenges were accompanied by renewed anxiety and helplessness, experienced as a phase of secondary despair, reflecting variability in adaptation trajectories across participants. Rejection by mainstream schools remains an unavoidable obstacle for many families. Although China’s education laws formally guarantee equal access to schooling for individuals with disabilities, some public institutions still refuse to admit children with autism. Even when enrollment is secured, these children may later face expulsion or peer discrimination. The educational environment is especially restrictive for children with moderate to severe autism (Huang, 2022).

“We’ve definitely been turned away, and not just once. It wasn’t just the primary school; whenever I tried to register for events, classes, or activities, as soon as they saw what kind of child he was, they’d find excuses to refuse me politely.” (Participant A7).

The difficulties that children with autism encounter in school enrollment stem largely from the limited inclusiveness of the education system and the gap between policy and implementation. Many schools hesitate to admit autistic children out of concern for teaching order and management capacity, while others lack the resources and professional support required for inclusive education. As a result, parents often struggle to secure stable school placements for their children, which generates persistent anxiety during the schooling process.

Even when enrollment is achieved, pronounced gaps in academic performance frequently bring renewed emotional distress. In a highly competitive social environment where academic achievement is closely tied to future employment and livelihood security, the absence of observable learning progress after sustained parental investment of time, energy, and money easily leads to frustration and despair. These reactions reflect not only disappointment with school performance, but also deeper anxieties about social realities and their children’s long-term prospects.

“When my child once ranked in the top 10, I believed he was truly improving and might become like other children. I became increasingly anxious and invested heavily in his school performance, but the more I invested, the greater the emotional blow. Eventually, I had to accept that he is not a high-achieving learner, but an ordinary child with autism.” (Interviewee A7).

After enrollment, the challenges of inclusion for autistic children began to surface. Schools accepting autistic students often require parents to accompany their child in class to mitigate certain risks. But once in the classroom, parents quickly notice the differences between their child and typical peers in behavior, learning, and social skills. These distinctions make parents acutely aware that autistic impairments are largely irreparable and that a “normal” life is unattainable.

“In primary school, you watch your child alongside typical children for at least 10 hours a day. You see how hard it is for him to play, eat, or interact. The realization makes you anxious all over again.” (Interviewee A1).

The pressures of education and inclusion can sharply undermine the hopeful expectations that parents of children with autism develop as their child makes progress, plunging them into a renewed state of despair that may be even deeper than that experienced at the initial diagnosis. Confronted with these challenges, parents begin to adopt divergent coping strategies, which in turn shape distinct adaptation trajectories.

#### 4.1.4 Divergent adaptation: active adaptation vs. adaptation difficulty

Parents of children with autism gradually diverge into distinct adaptation trajectories amid repeated setbacks. Some families engage in active strategy exploration and resource mobilization, reflecting active adaptation, whereas others experience persistent challenges, defined as adaptation difficulties. In the latter trajectory, ongoing frustration reduces expectations for further progress, limits perceived returns from effort, and leads to increasing withdrawal from social life. At the family level, marital, parent–child, and intergenerational relationships become fragile, leaving the household system vulnerable to varying degrees of tension (Lin et al., 2024; Lee et al., 2019). The use of neutral terminology highlights functional differences between trajectories without implying value judgments, capturing the complexity of families’ lived experiences.

“Each small improvement brought me joy, but every regression crushed me again. Seeing him sit beside typically developing children was profoundly painful. Sorrow, envy, and despair repeated themselves through endless cycles of collapse and recovery. Now, I feel numb.” (Interviewee A14).

The stage of adaptation difficulty should not be attributed to weak personal will, but to the cumulative suppression of protective factors by multiple risks. High rehabilitation costs, declining income, exclusionary educational and social environments, stigma, and the erosion of existing support networks jointly drain families’ social resources and deepen feelings of powerlessness and despair (Rusu et al., 2024). Under such conditions, withdrawal often emerges as a constrained response to prolonged structural pressure. At the same time, some parents actively seek new sources of support under pressure. By mobilizing available resources, adjusting daily routines, and regulating their emotional states, they gradually restore a degree of family stability.

“I keep telling myself that I cannot allow myself to be defeated. If I am defeated, everything is over. There would be no future for me and no future for my child. That would be truly tragic. I cannot give in to life. I have to keep solving problems.” (Interviewee A2).

Although the severity of a child’s impairment shapes daily caregiving demands, most parents emphasized that it does not explain the pronounced differences in family adaptation styles. As one parent noted, “Once a family is affected by autism, we are all in much the same position; any differences are a matter of degree rather than kind.” Nearly all respondents experienced comparable shock and helplessness at diagnosis, yet their adaptive trajectories gradually diverged through repeated setbacks. In this sample, only one child had mild ASD, while all others were moderate or severe. Differences in adaptation therefore appear to reflect variations in support systems and coping approaches rather than impairment severity itself.

### 4.2 Motivational sources of resilience for well-adapted parents

Well-adapted parents (*n* = 13) drew on three interrelated levels of motivational sources to foster resilience: individual, family, and societal. These sources acted as protective factors that buffered the effects of adversity and enabled positive adaptation, with each level reinforcing the others.

#### 4.2.1 Individual level: intrinsic traits of resilience

Resilience-related intrinsic traits form a relatively stable psychological core that enables individuals to recover from adversity and, in some cases, achieve post-traumatic growth. Among parents who adapt well, these traits are reflected in a strong sense of parental responsibility, firm personal conviction, heightened self-awareness, and a resilient, optimistic disposition. In particular, a deeply felt sense of parental responsibility prevents caregivers from abandoning their children even in moments of despair. Although legal norms impose a general obligation on all parents to care for their offspring, well-adapting parents are distinguished by a more deeply internalized moral motivation. The other-oriented belief that one must not fail one’s child often becomes a powerful inner force that drives parents to reconstruct their sense of agency and continue moving forward.

“As long as I am alive, I will do everything I can for my child. When the day comes that I close my eyes for the last time, I want to be able to tell myself that I did right by him and did not abandon him to suffer in society. If I had done that, I would never be able to rest in peace. I would not be a qualified father.” (Interviewee A7).

This deeply ingrained ethical responsibility within the parental role enables them to persist in caregiving duties and maintain basic family functioning even when facing profound adversity.

Second, unwavering belief sustains parents’ hope amid adversity. Belief under adversity is widely recognized as a vital internal resource for individuals to confront challenges and achieve positive adaptation (Shek, 2005). Though each autistic child and family faces unique circumstances, a steadfast, positive conviction consistently propels them through hardship, provides motivation for daily life, and constitutes a key component in the cultivation of individual resilience.

“At first, it was hard for me to accept. But after entering this community and meeting so many people, I came to realize that it was not the teachers or the doctors who ultimately healed these children, but a mother’s love. That realization made me believe that as long as I do my part, my child will get better.” (Interviewee A3).

Subsequently, the emergence of self-awareness helped parents adjust their mindset. Some parents struggling to adapt tend to completely identify with the caregiver role, leading to emotional strain and psychological burden. In contrast, parents who adapt better recognize their own needs, integrating both child-care and self-care into daily life.

“After the diagnosis, I lost myself for a long time. I felt that I existed only for my child. Later, when we accepted the reality, my husband and I realized that this would be a long journey and that we could not exhaust ourselves at the very beginning. We have to take care of ourselves first. If both of us are burned out, who will take care of our child in the future? So now I choose to accept the present and try to enjoy life.” (Interviewee A1).

Finally, resilient and optimistic dispositions strengthen parents’ capacity to cope with adversity. Prior research indicates that parents of children with autism who exhibit traits such as optimism, resilience, and extroversion tend to adapt more effectively and maintain more harmonious family relationships (Ma et al., 2015). This pattern is also evident in this study. Interviewee A2, for example, remained persistently optimistic throughout the intervention process and eventually supported her child in developing into a relatively accomplished young pianist. Although she experienced intense emotional distress following the diagnosis, her underlying optimistic outlook enabled her to regulate negative emotions and sustain positive interpretations during the long course of adaptation.

“I rarely fall into negative emotions because I am naturally optimistic. The first few years, especially the very first year, were undeniably painful. That pain lasted for at least a year and a half.” (Interviewee A2).

In sum, internalized parental responsibility, unwavering belief, emergent self-awareness, and a hardy–optimistic personality shield parents from stress and foster the development of resilience.

#### 4.2.2 Family level: interactive support as a resilience enabler

Family-level support plays a vital role in fostering resilience among parents of children with autism. Assistance from family members, together with the child’s occasional positive emotional feedback, reinforces parents’ motivation to confront adversity. Shared caregiving reduces parental stress, while mutual understanding, joint participation, and a stable emotional climate shape both treatment outcomes and parents’ perseverance (Xie et al., 2024). Within a family-oriented cultural context, such collective support activates individual resilience while alleviating overall parenting pressure (Han and Gao, 2025; Gao et al., 2023).

“Our family atmosphere is excellent. Grandparents on both sides have never blamed one another because of our child. When behavioral problems emerge, we discuss together how to help, and the whole family is united on this issue. That’s why I’ve never given up.” (Interviewee A13).

Spousal support constitutes one of the most critical forces within the family system. When parents of autistic children are engulfed by despair, the understanding and assistance of their partner provide the most immediate help, transforming the struggle from a solitary burden into a shared effort.

“In a family, after a major event occurs, your spouse’s attitude and actions greatly influence your own thoughts. If he or she isn’t supportive, it would make an already hard-to-accept situation feel even worse, like adding frost on top of snow.” (Interviewee A1).

Beyond assistance from adult family members, the child’s occasional emotional reciprocation also becomes an important source of strength that sustains parents’ endurance. Under long-term anxiety and fatigue, even a small warm response from the child can dissolve distress and reawaken parental motivation.

“Once I came home completely exhausted and [was in] no mood to talk. He asked me something, and I did not reply. Without a word, he came over, stood behind me, and began to massage my shoulders. I was stunned. I never expected anything in return, since caring for my child is a father’s instinct. After that first massage, I was so overwhelmed that I could not speak. He asked me again, ‘Dad, does it feel good?’ I could only say, ‘It feels incredibly good.’” (Interviewee A7).

Overall, family support systems are built upon the combined forces of assistance from household members, stable partner relationships, and emotional responsiveness from children. From practical caregiving to emotional reassurance, these elements form an interactive support network that enables parents to maintain motivation and emotional balance amid prolonged challenges.

#### 4.2.3 Societal level: external support as a resilience backbone

Diverse forms of social-level support provide crucial external resources for families affected by autism. Both informal and formal assistance help reduce caregiving burdens, while a more inclusive social climate facilitates community integration and strengthens parental resilience. Informal social support, in particular, plays a vital role in emotional regulation. Originating from daily interpersonal relationships outside the family, it offers companionship and understanding during emotional distress, alleviates feelings of social isolation, fosters a sense of acceptance, and thereby smoothens parents’ participation in community life.

“For example, my friends take me out for a meal or go shopping with me, and his friends invite him to play basketball. They may not see this as ‘help,’ but this kind of companionship and social interaction really helps lift my mood.” (Interviewee A12).

“Because I attend classes with my child, whenever I encounter a problem that I cannot resolve on my own, I discuss it with the teachers and we work on it together. They offer suggestions and then implement joint interventions at the center.” (Interviewee A5).

Second, formal social support provides parents with institutional safeguards. This support originates from government agencies and voluntary service organizations. Due to shared circumstances, many parents of autistic children have spontaneously formed parent-led mutual-aid organizations. These organizations take it as their responsibility and mission to provide a safe space and social support for families navigating autism. Among the formal support available to families dealing with autism, these organizations deliver the most direct and convenient help, supplying both instrumental and emotional aid at the moments parents need it most.

“Parent organizations have been immensely helpful to me. Through activity organization and group discussions, parents exchange experiences and identify shared problems. Whenever I encounter difficulties, such as choosing appropriate intervention institutions, I seek advice in the parent groups and receive many practical suggestions. This form of support is far more direct and convenient than seeking help through other channels.” (Interviewee A11).

Government agencies, as the primary welfare actors for disadvantaged and special-needs households, play a vital role in formal social support accessible to the autism community. Recognizing the rising prevalence of autism and the widespread hardship it causes, authorities have introduced a range of protections that lighten family burdens and create conditions conducive to children’s rehabilitation and development.

“Our subsidies come from several sources. The Disabled Persons’ Federation provides an annual medical grant of 16,000 RMB (about USD 2,200) for children with autism who require intervention, usable at designated centers. There is also a monthly living allowance of several thousand RMB. In addition, the sub-district and community provide annual subsidies for households with disabled members, equivalent to about 1,800 RMB per month for a couple (roughly USD 250). These constitute the main forms of policy-based financial support currently available to such families.” (Interviewee A7).

Whether informal support from friends and professionals or formal support from parent-led mutual-aid organizations and government agencies, these resources alleviate caregiving pressure, stabilize families’ support systems, and enhance parents’ coping capacity. The inclusivity of the social environment further shapes family adaptation by reducing discrimination and facilitating social integration. By contrast, exclusionary environments intensify both children’s functional difficulties and parental stress, discouraging participation and weakening resilience. As key micro-level social settings, schools vividly reflect this dynamic: biased school cultures hinder rehabilitation and magnify distress, whereas inclusive environments promote children’s skills and family integration.

“When he was in the fourth grade, he met a music teacher who treated him with great encouragement. She praised him in every class and invited him to perform on stage. My child changed completely. He even said, ‘This is the best teacher I have ever had.’” (Interviewee A2).

The community serves as a crucial everyday space for the development of children with autism and their families (Edmunds et al., 2025). An inclusive community environment enhances families’ sense of belonging and facilitates social integration. For example, B5 once caused a neighborhood misunderstanding due to difficulties in self-expression when he impulsively grabbed a classmate’s wrist while attempting to add the child as a friend via a smartwatch. After the incident was shared in a neighborhood group chat, Interviewee A5 apologized and explained the situation, expecting reproach. Instead, neighbors responded with understanding and comfort, which significantly alleviated parental anxiety and strengthened the family’s sense of social acceptance and integration.

“All the neighbors in our group comforted me and said, ‘You really have it tough.’ They told me that now they understood the situation and would know how to interact with him in the future. I was deeply moved by their kindness.” (Interviewee A5).

Overall, the external support system, composed of diverse social support and an inclusive social environment, provides a sustainable source of momentum for autism-affected families, effectively enhancing their resilience amid adversity and offering vital backing for positive family adaptation and development.

Motivational sources at the individual, family, and societal levels do not operate in isolation. Rather, they form a dynamic cycle through the interaction between psychological capital and resource acquisition. Psychological capital, including parental responsibility, beliefs, self-awareness, and optimism, provides the initial impetus for resource acquisition, prompting parents to engage with family networks, patient support groups, and institutional resources. The outcomes of resource acquisition then either reinforce or diminish psychological capital, producing either self-reinforcing positive accumulation or self-defeating negative depletion cycles. Previous research demonstrates a higher-order structure of character strengths, including a G-factor (good character) and three S-factors (self-control, inquisitiveness, and caring), which significantly influence well-being (Li et al., 2022). This cyclical interaction provides an explanatory framework for the emergence and divergence of adaptation trajectories among parents of children with autism.

### 4.3 Adaptive strategies of well-adapted parents

Well-adapted parents employed three core adaptive strategies that were closely linked to their motivational sources of resilience. These strategies were dynamic and context-dependent, and were used in combination to cope with the diverse challenges of ASD caregiving.

#### 4.3.1 Active self-adjustment: cognitive and emotional regulation

When life circumstances change dramatically, individuals often restore internal equilibrium through self-adjustment, gradually aligning emotions and behaviors with new environmental demands (Riepenhausen et al., 2022). For parents of children with autism, resilience fosters proactive self-regulation amid adversity, typically manifested through enhanced autism literacy and reconstructed meaning in life.

First, a deepening understanding of autism serves as the starting point for family adaptation. Influenced by entrenched beliefs and social biases, many parents struggle to genuinely comprehend and accept their child due to knowledge gaps, leading to persistent denial and resentment. Better-adapted parents actively seek information about autism, enabling them to view their child’s characteristics through a more realistic and calmer lens and gradually cultivating acceptance and inclusiveness.

In the immediate aftermath of diagnosis, Interviewee A5 was devastated and initially unable to accept that her child had autism. Yet, driven by responsibility and familial bonds, she moved beyond denial to systematically study autism-related knowledge. As her understanding deepened, she gradually overcame her initial fear of viewing autism as a severe defect and ceased attributing all issues to her child or herself. Instead, she began engaging with her child’s traits through a more peaceful approach.

“At first, I still held on to some unrealistic hopes. After I resigned and began to learn how to care for him, those years of living and working with him every day gradually increased my sense of acceptance. I truly felt that his condition had improved a great deal.” (Interviewee A5).

Proactive learning about autism after diagnosis helps parents reinterpret their child’s behavioral logic. Many acts that appear “non-compliant” or “difficult to manage” in fact stem from sensory hypersensitivities or atypical neurodevelopment rather than deliberate defiance. This cognitive shift reduces misinterpretation and mitigates parenting frustration. As knowledge expands, parental fear and uncertainty subside, acceptance of the child increases, and emotional stability improves, facilitating smoother parent–child interaction and enhancing caregivers’ sense of efficacy.

Second, the construction of positive meaning functions as a crucial psychological coping strategy for parents confronting adversity. Faced with the uncertainty of long-term caregiving, parents reinterpret their experiences to restore a sense of order in life. Interviewee A4 illustrates this process: during his child’s diagnosis period, his business unexpectedly recovered from a downturn, which he later interpreted as a meaningful turning point linked to his child’s misfortune. Although this interpretation lacks objective causality, it alleviated his emotional distress and enabled the family to approach the child’s condition with greater gentleness and acceptance. For many families dealing with autism, meaning-making is not a denial of reality but a way of sustaining psychological strength under prolonged pressure.

“Later, I told my wife that perhaps our child’s condition had, in a way, protected the family from other misfortunes, and she came to agree. Since then, no one else in the family has faced serious illness, and our business has also improved.” (Interviewee A4).

Overall, self-adjustment emerges as the most sustainable adaptation strategy among parents of children with autism. Unlike reliance on external resources, it can be continuously adjusted to changes in the child’s condition and family rhythms. As parents undergo internal shifts in attitudes, emotions, and cognition, parent–child interactions become smoother, family dynamics stabilize, and feelings of anxiety and helplessness gradually diminish. For many families, self-adjustment not only enables them to endure the most difficult periods but also helps restore everyday order and forward momentum in life.

#### 4.3.2 Building diverse social support networks: mobilizing internal and external resources

Beyond self-adjustment, parents mobilize social resources to build broader support networks that strengthen their capacity to cope with adversity. Within the family, a stable marital relationship constitutes a crucial pillar of resilience, as spouses’ ability to rebuild emotional connection directly shapes both the family climate and caregiving outcomes. The experience of Family B5 illustrates this process. After diagnosis, pre-existing personality differences between the parents were amplified, and prolonged conflict pushed the household to the brink of collapse, which hindered the child’s rehabilitation. In response, the couple set aside daily time for communication and reflection, gradually adjusted their interaction patterns, and ultimately restored household stability, accompanied by noticeable improvement in the child’s condition.

“Seeing his changes pushed me to reflect on my own problems. During that time, I read extensively on psychology, trying to understand how our different family backgrounds had shaped our conflicts, and adjusting my own behavior. My changes triggered positive responses from him, and gradually we improved together, entering a virtuous cycle that allowed us to cope with our child’s challenges more effectively.” (Interviewee A5).

The improvement in the couple’s relationship brought more than emotional relief. Smoother communication enabled both partners to better understand caregiving pressures, express stress in time, and coordinate the division of responsibilities more effectively. A stable partnership thus became a key source of endurance for the family. Family A1 illustrates this process: as the couple’s communication improved, household roles became clearer, daily care grew more orderly, and the child’s intervention could proceed without disruption.

“I was responsible for overall planning, while the grandparents managed the household. During the intensive rehabilitation period, I continued working, so my father-in-law handled transportation and my mother-in-law took care of daily chores. My husband focused on earning income. He initially tried to study the condition on his own, but later realized this was unsustainable because the household still needed to function. That is how our current division of labor gradually took shape.” (Interviewee A1).

Owing to the distinctive challenges of autism, these families are especially vulnerable to disruptions in relationships, daily functioning, and emotional climate. While internal family support provides essential emotional and material assistance, it is often insufficient for sustained long-term adaptation. Parents therefore increasingly rely on external resources, among which parent-led mutual-aid organizations have become a key channel for systematic support. Drawing on her educational background and awareness of service gaps, Participant A1 co-founded such an organization to build a shared caregiving community: “The autism journey is too difficult to walk alone, so we wanted parents to walk it together.” Since its establishment, the organization has regularly facilitated experience sharing and disseminated policy and service information.

“Every July and August, we organize parent sharing sessions to exchange experiences related to children’s rehabilitation and growth. New resources and policies are shared in our group chat, and we sometimes invite experienced parents from other regions to offer guidance. We do this not for personal gain, but to give parents a sense of belonging.” (Interviewee A1).

Simultaneously, most parents join parent-led mutual-aid organization to bolster their social networks. Within these organizations, parents gain emotional support through recognition and understanding from peers, informational support through advice and guidance, and occasional material assistance, all of which help them resolve crisis situations promptly.

“I mainly take part in the flower-arranging sessions, which I find the most enjoyable. There are also tea-tasting outings and outdoor activities organized for parents. These activities function like group-healing spaces. While participating in them, I can temporarily stop thinking about my child and all the distress associated with caregiving, and I feel deeply relaxed.” (Interviewee A11).

In addition, connecting with university volunteers constitutes another important way to diversify support networks. College students, with their learning capacity, empathy, and, in some cases, professional skills, can provide companionship, training, and social-participation opportunities for children with autism, while also easing parental caregiving pressure. Recognizing these advantages, Interviewee A7 co-founded the “Xingyue Integration” volunteer team with other parents and established long-term collaborations with multiple universities in Wuhan. Through these sustained partnerships, both him and his child were able to expand their social networks and access stable external support.

“They are an important part of my social support system. Compared with younger volunteers, university students have greater maturity and social experience, and they know better how to interact with children like mine. That is why I actively seek to work with them in many activities.” (Interviewee A7).

Building social support positively impacts parents of children with autism in adapting to challenges. On one hand, it helps reduce negative emotions and maintain mental health. On the other hand, it strengthens their social support network, enhancing their resilience in coping with difficulties.

#### 4.3.3 Proactive social integration: advocating for inclusivity for families of children with ASD

Fearing public prejudice and discrimination, most parents of children with autism are reluctant to enter the mainstream spotlight. In contrast, well-adapted parents motivated by adaptive forces employ not only self-adjustment and social-support strategies but also inclusion tactics that encompass school, community, and social integration. This proactive integration seems to symbolize their genuine resistance to adversity.

First, successful school enrollment and shared development with typically developing peers represent a core aspiration for many families of children with autism, yet achieving this goal often requires close collaboration between parents and schools. From parents’ perspectives, emotional communication, relationship building, and proactive resource linkage are key strategies for facilitating school entry and social integration. Interviewee A7 exemplifies this process. Before enrollment, he contacted the school principal directly and, with support from a staff member at the Disabled Persons’ Federation, secured his child’s admission. Afterward, he maintained regular communication with the homeroom teacher to obtain necessary support and joined the class parent committee to participate more actively in school affairs and future activity organization.

“My organization is affiliated with the Jiang’an District Disabled Persons’ Federation. Through regular contact, I became acquainted with a section chief and often shared my views on school inclusion. Over time, he came to support these ideas, and later assisted us with my child’s school enrollment.” (Interviewee A7).

After school enrollment, joint activities become an important means of promoting the social integration of children with autism into typically developing peer groups. Shared extracurricular experiences create opportunities for interaction that foster mutual understanding and acceptance between autistic and non-autistic children. The “Xingbao Public-Welfare” initiative launched by Interviewee A6 exemplifies this approach, providing a sustained setting in which children complete tasks and play together. A6 further noted that the inclusive attitudes developed among typically developing children often extend to their families, gradually shaping more positive perceptions of autism in the wider social environment.

“The inclusive climate at our school has been weak since my child entered first grade. I believe it is the parents’ responsibility to facilitate school-child communication. To promote classmates’ understanding of autism, I invite them to join our Xingbao public welfare activities, where direct participation helps foster acceptance.” (Interviewee A6).

Secondly, community integration serves as a vital pathway for autistic children’s social inclusion, providing a crucial platform for social interaction. Participating in community activities offers a straightforward and effective way for autistic families to integrate into their neighborhoods. For parents of autistic children, engaging in community events is not only a means to access support but also a form of self-empowerment and social connection, moving them from isolation to connection.

“I am willing to share my child’s situation with my neighbors, partly to help them gradually understand autism, and partly because once they know him better, they become more tolerant and even willing to guide him on what is appropriate. I believe this benefits both my child and our family. By exposing him to these situations while he is still young, society is more likely to accept him, and he can slowly learn how to interact with others properly.” (Interviewee A5).

Finally, compared to school and community settings, social integration poses a greater challenge for families dealing with autism. They address this by proactively seeking resources and actively creating inclusive environments. Interviewee A1 collaborates with social-welfare advocates, arranging interactions between autistic children and other disadvantaged groups to foster social understanding and adaptation, thereby achieving deeper inclusion.

“There is an ‘Angel Sign-Language Restaurant’ in Wuhan that employs deaf and hard-of-hearing staff. My child had previously attended basic sign-language volunteer training, so I took him there to experience the environment and communicate through sign language. During that activity, his performance was noticeably better than usual. Normally in shops he just wants to run around and cannot sit still.” (Interviewee A1).

In essence, integration into schools, communities, and broader social life constitutes the concrete pathway through which families of children with autism pursue social inclusion in everyday contexts. The goal of integration is not to require autistic children to fully conform to mainstream norms, but to enable participation in social life through mutual adjustment and understanding across different developmental stages. This process depends both on parents’ sustained efforts and on stable support from schools, communities, and public systems. Only through the joint engagement of all parties can social understanding and acceptance of autism continue to expand, allowing these families to occupy more secure and dignified positions in public life.

## 5 Discussion

### 5.1 Key findings: dynamic resilience and culturally specific adaptive processes

Through in-depth interviews with 16 parents of children with autism, this study captures the complex life conditions and emotional fluctuations experienced by families during long-term caregiving. Parental adaptation does not occur in a linear or fixed manner; instead, it unfolds through repeated shifts among phases of initial collapse, emerging hope, and subsequent setbacks. As children progress through stages such as diagnosis, intervention, school enrollment, and social participation, parents’ daily routines, emotional states, and coping strategies adjust accordingly. Resilience theory provides a useful framework for understanding these processes; however, participants’ ability to sustain resilience appears to depend on personal orientations, available family support, and the inclusiveness and accessibility of social resources.

The burdens experienced by families are embedded in broader structural conditions. Parents’ challenges arise not only from emotional strain and long-term caregiving demands, but also from limited institutional support, uneven distribution of social resources, and gaps in educational and medical systems. In seeking treatment, schooling, and opportunities for social participation, parents often encountered weak policy implementation, fragmented service provision, and restricted resource accessibility, leading them to rely heavily on household assets and personal networks. Within this context, family adaptation trajectories diverged. Some parents developed relatively stable coping mechanisms through ongoing trial and adjustment, while others remained constrained under sustained pressure. Many parents gradually rebuilt daily routines within prolonged adversity by learning about autism, maintaining essential self-care, expanding support networks, and participating in social activities when feasible. Through repeated experimentation and reflection, they identified more sustainable ways of managing everyday life. Their resilience was not inherent, but emerged from iterative trials and ongoing negotiation with daily challenges.

At the institutional level, despite the introduction of support policies for families with children with autism, substantial gaps remain in implementation, resource allocation, and regional equity. Participants’ accounts indicate that the difficulties faced by families cannot be reduced to individual or household issues, but are intertwined with limited public awareness, inadequate institutional support, and restricted access to social resources. Policy improvements should be focused on strengthening interdepartmental coordination, developing effective inclusive-education systems, ensuring continuity and quality of rehabilitation services, and establishing long-term care support mechanisms.

### 5.2 The cycle–divergence model: a theoretical framework for parental coping in ASD

Through in-depth interviews with 16 parents, in this study we identify a “cycle–divergence” adaptation trajectory. After their child’s diagnosis, parents typically experience an initial breakdown characterized by psychological shock and uncertainty. During the early intervention phase, many achieve preliminary adaptation, gaining confidence through small but meaningful developmental gains. As children enter the education and inclusion phase, parents often face renewed challenges, which we describe as “secondary despair,” related to school access, social integration, and institutional constraints. At this point, parents diverge into distinct functional states that reflect how their adaptation unfolds over time.

These functional states are shaped by the dynamic interaction between psychological capital, including parental responsibility, beliefs, self-awareness, and optimism, and resource acquisition, such as family support, social networks, and institutional connections. In the accumulation cycle, parents who maintain confidence and proactive engagement make use of available resources, which in turn reinforces their sense of efficacy and resilience. Conversely, in the depletion cycle, limited access to resources or negative experiences diminish psychological capital, reducing the ability to seek support and amplifying stress. These dynamics give rise to two observable patterns. The resource-expansion type is characterized by active problem-solving, outward social engagement, and a strengthened sense of capability. The resource-contraction type is marked by withdrawal, reduced relational engagement, and a growing sense of powerlessness. These patterns emerge from structural interactions rather than individual moral choices, highlighting that adaptation is co-shaped by personal and environmental factors.

The counterintuitive phenomena revealed in this study, such as “initial adaptation” and “secondary despair,” challenge the assumption of linear progression implicit in Pressman (1987) five-stage model and Gou (2024) three-stage model. Adaptation is reconceptualized as a cyclical, fluctuating process (cyclical adaptation) triggered by institutional factors, showing that institutional turning points have greater psychological impact than natural developmental stages. Building on this, we expand the resilience perspective proposed by Greeff and van der Walt (2010), shifting focus from static individual traits or closed family systems to the interactive cycle between psychological capital and resource acquisition. Our findings complement prior research on ASD parenting by highlighting dynamic adaptation processes and the interplay between personal, familial, and structural resources.

Individual strengths, such as character strengths, and positive experiences, including flourishing, support the development of resilience (Bu and Duan, 2021). Importantly, this model is based on a sample of urban, resource-rich families in Wuhan, and the identified mechanisms may vary in other contexts. Nevertheless, the cycle–divergence framework provides a nuanced explanation of how personal resources, social support, and structural conditions interact to shape adaptation trajectories among parents of children with autism. This approach highlights the critical role of relational negotiation and structural factors in parental coping, offering a contribution that goes beyond individual trait-focused resilience models and emphasizing the context-dependent nature of adaptation processes.

### 5.3 Implications for inclusive positive psychology interventions and policy

The findings of this study have important implications for the design and implementation of inclusive positive psychology interventions for parents of children with ASD (a key vulnerable group) and for policy support in China and other low- and middle-income countries with similar cultural and resource contexts.

#### 5.3.1 Implications for positive psychology intervention practice

Given the cyclical, stage-based nature of parental adaptation to ASD, interventions may be tailored to the specific challenges observed at each stage. For instance, trauma-informed counseling can address the post-diagnosis “collapse and despair” phase, skill-building activities may support parents during the “initial adaptation” phase, and school inclusion support can assist families experiencing “secondary despair.”

Interventions should foster culturally specific resilience mechanisms by centering on the strengths of Chinese families, such as family collectivism and relational social support. Parent training workshops may strengthen family caregiving systems and enhance spousal and intergenerational support, while group interventions can leverage parent mutual-aid organizations, as reported by participants, to facilitate peer support and collective adaptation.

Interventions may also integrate components that promote active adaptation, including enhancing autism literacy and meaning-making capacities through ASD knowledge workshops and narrative therapy. These strategies help parents progress from initial denial to acceptance and reconstruct positive life meaning, based on challenges and coping strategies identified in participants’ narratives.

#### 5.3.2 Implications for policy and institutional support

Strengthening of financial and institutional support for families of children with ASD. The Chinese government should increase financial subsidies for ASD intervention and expand access to free or low-cost rehabilitation services, particularly for low-income families. Policies should also be implemented to enforce the right of children with ASD to attend mainstream schools and to provide training for schoolteachers on inclusive education for children with ASD.

Support for the development of parent-led mutual-aid organizations. The government and civil society organizations should provide funding, training, and institutional support for parent-led ASD mutual-aid organizations, which are a critical source of social support for parents and a key platform for inclusive positive psychology interventions.

Promotion of ASD awareness and reduce social stigma. Public education campaigns should be launched to increase ASD awareness and foster an inclusive social environment for families of children with ASD. These campaigns should target schools, communities, and the general public to reduce discrimination and improve social integration for children with ASD and their parents.

### 5.4 Limitations and future research

This study has several limitations that should be acknowledged, which also point to directions for future research. The sample primarily consists of urban parents in Wuhan with a relatively high level of education and certain economic and social resources, with a low proportion of male caregivers (only three fathers). As a result, the findings mainly reflect the coping experiences of urban, well-resourced families and may not capture the full range of experiences among all parents of children with autism. In particular, rural families facing geographical isolation and limited access to services, parents with lower educational attainment, low-income families, and fathers serving as primary caregivers may be underrepresented. These limitations suggest that the mechanisms identified in this study, such as the interaction between psychological capital and resource acquisition, should be interpreted cautiously in different social or economic contexts. Future research should examine whether the cycle–divergence adaptation model applies to more diverse family backgrounds, in order to assess its generalizability.

While the study traces parental adaptation across different stages through retrospective interviews and observation, it is a cross-sectional study and cannot capture long-term longitudinal changes in resilience and adaptation. Future research should adopt a longitudinal design to examine the dynamic changes in parental resilience over time and the factors that shape these changes.

This study focuses on parental resilience and adaptation, but it does not examine the bidirectional relationship between parental resilience and the child’s ASD development. Future research should explore how parental resilience influences child outcomes and how child outcomes, in turn, shape parental resilience—an important direction for family-centered positive psychology interventions.

Despite these limitations, this study provides important empirical evidence for the resilience and adaptive processes of parents of Chinese children with ASD and contributes to the global evidence base of inclusive positive psychology interventions for vulnerable caregivers.

## Data Availability

The raw data supporting the conclusions of this article will be made available by the authors, without undue reservation.
